# Evaluation of Pencycuron Residue Dynamics in Eggplant Using LC-MS/MS and Establishment of Pre-Harvest Residue Limits

**DOI:** 10.3390/foods13233754

**Published:** 2024-11-23

**Authors:** Da-Geon Lee, Jae-Woon Baek, Hye-Ran Eun, Ye-Jin Lee, Su-Min Kim, Tae-Gyu Min, Yong-Won Cho, Yoon-Hee Lee, Yongho Shin

**Affiliations:** Department of Applied Bioscience, Dong-A University, Busan 49315, Republic of Korea; ekrjs1221@donga.ac.kr (D.-G.L.);

**Keywords:** pencycuron, eggplant, graphitized carbon black, LC-MS/MS, residue dynamics, maximum residue limit, pre-harvest residue limit, food safety

## Abstract

Pencycuron is a fungicide whose maximum residue limit (MRL) in eggplant is either set at very low levels (0.02 mg/kg in European Union) or remains unestablished in many countries, necessitating stringent pesticide management. To enable timely interventions by farmers and regulators, pre-harvest residue limits (PHRLs) propose maximum allowable pesticide concentrations for each day during the pre-harvest period. An analytical method was developed to conduct residue determination trials, demonstrating that graphitized carbon black (GCB) effectively removes eggplant matrices during sample preparation. The LC-MS/MS method was established with a limit of quantification (LOQ) of 0.005 mg/kg, recovery rates ranging from 102.6% to 106.1% with relative standard deviation (RSD; 2.3–6.4%), and a matrix effect (%ME) of +8.1%. Residue analysis revealed a concentration of 0.045 mg/kg at 0 days after treatment (DAT), decreasing to 0.006 mg/kg at 14 DAT. The residue dynamics followed a first-order kinetic model, as confirmed by the F-test, with a rate constant of 0.1405. Therefore, the half-life was determined to be 4.9 d. Based on the MRL value of 0.02 mg/kg at 0 days before harvest (DBH), the PHRL was determined using both *k* and *k*_min_, resulting in values of 0.04 mg/kg and 0.02 mg/kg at 5 days and 0.08 mg/kg and 0.03 mg/kg at 10 DBH, respectively. Using *k*_min_ yields more conservative results, which ensures food safety under conditions of slower degradation rates.

## 1. Introduction

**Figure 1:**
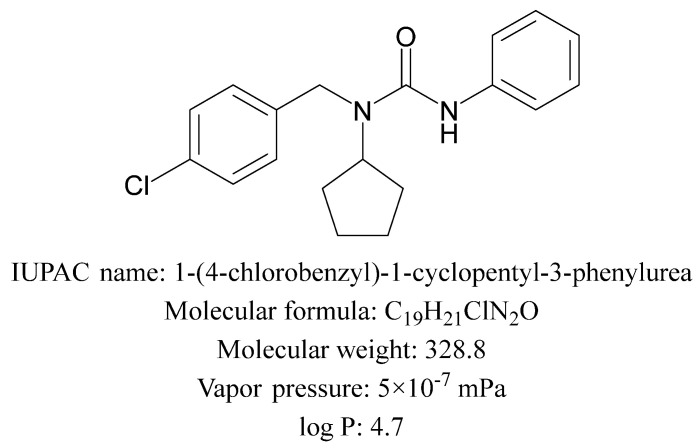
IUPAC name, structural formula, and physicochemical properties of the fungicide pencycuron.

Pencycuron, systematically named 1-(4-chlorobenzyl)-1-cyclopentyl-3-phenylurea (Figure 1), is a non-systemic fungicide classified under the phenylurea chemical family. It has been extensively adopted in agricultural settings due to its effectiveness in combating a broad spectrum of fungal diseases. Notably, pencycuron demonstrates high efficacy in managing black scurf in potatoes, sheath blight in rice, and damping-off in ornamental plants [1]. The fungicidal action of pencycuron is primarily achieved through the inhibition of cell division and the disruption of β-tubulin assembly, which collectively impede the growth of fungal mycelia [2,3]. This precise mechanism of interfering with fungal cellular processes makes pencycuron an essential tool for controlling plant diseases that can adversely affect crop yield and quality [4,5].

The prolonged environmental persistence and abnormal residual presence of pesticides on farmland can significantly elevate health risks for consumers [6]. These residues may accumulate in agricultural soils and products, leading to exposure through the food chain [7]. To accurately assess the risks associated with pesticide exposure, it is essential to conduct residue studies that examine the dynamics of pesticide residues in agricultural products [8]. Such studies, conducted worldwide, enable the determination of safe residue limits and inform regulatory standards to strengthen food safety in each country [9]. Therefore, investigating the residue dynamics of pesticides like pencycuron is essential for establishing safe agricultural practices and protecting public health.

To manage the risks associated with agrochemical residues, regulatory frameworks such as the maximum residue limit (MRL) have been established [10]. The MRL represents the allowable limit of pesticide residues in crops at the point of harvest and consumption, serving as a critical control point before agricultural products reach consumers. If these limits are exceeded, actions such as postponement of shipment, alteration of usage, or disposal of the affected produce are implemented to prevent contaminated products from entering the market [10].

Pre-harvest residue limits (PHRLs) are particularly important for predicting and managing the possibility of exceeding the MRL between the final pesticide application and the pre-harvest interval (PHI) [11]. By proposing maximum allowable pesticide concentrations for each day during the pre-harvest stage—typically starting ten days before the scheduled shipment date—PHRLs enable farmers and regulators to take timely actions [12]. Establishing a reliable residue reduction prediction model involves calculating the degradation regression equation and the half-life of residual pesticides [13,14]. This approach allows for a reasonable forecast of residue decline, ensuring that the levels remain within safe limits as the harvest date approaches [15].

Eggplant (*Solanum melongena* L.), a member of the Solanaceae family, is a widely cultivated vegetable prized for its versatility, nutritional benefits, and significant economic value, especially in tropical and subtropical regions. The health benefits of eggplant are largely attributed to its high phenolic content. These phenolic compounds have demonstrated the ability to inhibit intestinal α-glucosidase, an enzyme involved in glucose absorption, thereby reducing glucose uptake and helping to manage hyperglycemia [16]. Additionally, they offer protective, anti-aging effects that contribute to the prevention of cardiovascular diseases and skin ailments caused by ultraviolet exposure [17]. Eggplant is highly susceptible to pests and fungal diseases, necessitating regular applications of pesticides, including insecticides and fungicides, to ensure healthy yields and high-quality produce [18]. The intensive use of these pesticides presents challenges in managing residual pesticide levels. The European Union (EU) has established set limits for pencycuron in eggplant to ensure consumer safety, establishing an MRL of 0.02 mg/kg [19]. The Republic of Korea has not set a specific MRL for pencycuron in eggplant, creating a regulatory gap that leads to uncertainty among farmers regarding safe usage levels. This lack of regulation poses risks of non-compliance with international residue standards, particularly in export markets with strict enforcement. Therefore, it is important to verify that pencycuron residues in eggplant do not exceed the MRL after the PHI, and the establishment of daily PHRLs through field studies is essential.

This study aims to evaluate the residue levels of the fungicide pencycuron in eggplants using liquid chromatography-tandem mass spectrometry (LC-MS/MS) and to establish reasonable PHRL. The research focuses on developing and validating a highly sensitive and accurate LC-MS/MS method, including the optimization of sample preparation procedures, for the reliable detection of pencycuron residues in the samples. Residue studies under greenhouse cultivation conditions were conducted to determine the degradation patterns of suspension concentrate (SC) formulation in eggplants and calculate its half-life. Based on these findings, we provided scientifically based recommendations for safe pesticide residue levels at harvest time, thereby ensuring food safety and regulatory compliance.

## 2. Materials and Methods

### 2.1. Chemicals and Reagents

Pencycuron standard (98.7%) was sourced from Sigma-Aldrich (St. Louis, MO, USA). Acetonitrile (HPLC grade) was purchased from Thermo Fisher Scientific (Waltham, MA, USA). Methanol (LC-MS grade) was obtained from Thermo Fisher Scientific (Waltham, MA, USA). Acetone (HPLC grade) was purchased from Duksan Pure Chemical (Seoul, Republic of Korea). LC-MS grade water was obtained from Merck (Darmstadt, Germany). Formic acid (>99%) was sourced from Thermo Fisher Scientific (Waltham, MA, USA). QuEChERS partitioning salts (4 g magnesium sulfate MgSO_4_, 1 g sodium chloride NaCl, 1 g trisodium citrate dihydrate Na_3_C_6_H_5_O_7_·2H_2_O, and 0.5 g disodium hydrogen citrate sesquihydrate Na_2_HC_6_H_5_O_7_·1.5H_2_O) were supplied by Chiral Technology Korea (Daejeon, Republic of Korea). Dispersive Solid Phase Extraction (dSPE) sorbents containing 150 mg MgSO_4_ and 25 mg primary and secondary amine (PSA) were obtained from BEKOlut (Haupststuhl, Rhineland-Palatinate, Germany), and dSPE sorbents containing 25 mg PSA, 7.5 mg graphitized carbon black (GCB), and 150 mg MgSO_4_ were purchased from Agilent Technologies (Santa Clara, CA, USA). The pesticide product, Monceren (25% SC), was provided by Farmhannong (Seoul, Republic of Korea).

### 2.2. Preparation of Working Solutions and Matrix-Matched Standard Solutions

Pencycuron analytical standard was dissolved in acetone to generate a stock solution at a concentration of 1000 mg/L. This solution was subsequently diluted with acetonitrile to obtain working solutions at concentrations of 10, 5, 2.5, 1, 0.5, 0.25, 0.1, 0.05, 0.025, 0.01, and 0.005 mg/L. These concentrations were used for the preparation of the solvent standard calibration curve and for fortification in the recovery experiments. For the preparation of matrix-matched standard solutions, 350 μL of the extract from a pesticide-free eggplant sample was combined with 350 μL of each working solution in a 5:5 ratio. The resulting concentrations of matrix-matched standard solutions used for calibration curve construction were 0.25, 0.125, 0.05, 0.025, 0.0125, 0.005, and 0.0025 mg/L. This corresponds to an analyte concentration of 0.5 to 0.005 mg per kilogram of sample.

### 2.3. LC-MS/MS Instrumental Condition

Instrumental analysis was conducted using an LCMS-8040 triple quadrupole mass spectrometer (Shimadzu, Kyoto, Japan) coupled with a Nexera liquid chromatograph (Shimadzu, Kyoto, Japan). Chromatographic separation was performed on a Kinetex PS C18 column (2.6 µm particle size, 3 × 100 mm; Phenomenex, Torrance, CA, USA) maintained at 40 °C. The mobile phase consisted of (A) water containing 0.1% formic acid and (B) methanol containing 0.1% formic acid. The gradient program for (B) was as follows: 0–0.2 min, 60%; 0.2–0.5 min, 20%; 0.5–5 min, 2%; 5–8 min, 2%; 8–8.5 min, 60%; and 8.5–12.5 min, 60%. The flow rate was set at 0.2 mL/min, and the injection volume was 2 µL.

The mass spectrometer was operated in electrospray ionization positive mode (ESI+). The nebulizing gas flow was set at 3 L/min, and the drying gas flow at 15 L/min. The desolvation line (DL) temperature was maintained at 250 °C, and the heat block temperature at 400 °C. Collision-induced dissociation (CID) was performed using argon gas with a purity of 99.999%. Data processing was performed using Shimadzu LabSolutions software (version 5.120).

### 2.4. Field Trials of Pencycuron Application on Eggplants

Field trials were conducted in Sancheong-gun, Gyeongsangnam-do, Republic of Korea, using eggplants (variety ‘Auto King’) as the test crop under greenhouse cultivation conditions, where temperatures ranged from 12.0 °C to 46.2 °C, and relative humidity levels varied between 58.7% and 91%. Pencycuron agrochemicals were applied as a foliar spray at a dilution rate of 2000 times. The pesticide was administered using a Perfect EL sprayer (Model EL 969-2; Siheung, Republic of Korea) at an application volume of 1800 L/h. Applications were made three times at seven-day intervals. Following the final application, samples were harvested at 0, 3, 5, 7, and 14 days after treatment (DAT). Collected samples were individually placed in labeled kraft paper bags to prevent cross-contamination and were immediately transported to the laboratory. The eggplant samples, with leaves removed, were uniformly ground using a blender with the addition of dry ice. The homogenized samples were stored at –20 °C until further analysis.

### 2.5. Sample Preparation

The sample preparation method was a modified version of the QuEChERS EN-15662 method [20]. A 10 g portion of the sample was weighed into a 50 mL centrifuge tube. Acetonitrile (10 mL) was added, and the mixture was shaken at 1300 rpm for 2 min using a shaker (1600 MiniG, SPEX SamplePrep, Metuchen, NJ, USA). QuEChERS salts consisting of 4 g MgSO_4_, 1 g NaCl, 1 g Na_3_C_6_H_5_O_7_·2H_2_O, and 0.5 g Na_2_HC_6_H_5_O_7_·1.5H_2_O were added to the tube. The mixture was shaken again at 1300 rpm for 2 min. The sample was centrifuged at 3500 rpm for 5 min using a centrifuge (Model 1248, LABOGENE, Lillerød, Denmark). The organic upper layer (1 mL) was then transferred to a dSPE tube containing 25 mg PSA, 7.5 mg GCB, and 150 mg MgSO_4_, and gently agitated for 1 min. The tube was centrifuged at 13,000 rpm for 5 min using a microcentrifuge (M15R, Hanil Scientific, Gimpo, Republic of Korea). A 350 μL aliquot of the supernatant was mixed with 350 μL of acetonitrile, and 2 µL of this mixture was injected into the HPLC-MS/MS system for the analysis of pencycuron.

### 2.6. Analytical Method Validation

The analytical method was validated for limit of quantification (LOQ), linearity of calibration curve, recovery, and matrix effect in accordance with standard guidelines for pesticide residue analysis. The LOQ was established as the lowest concentration of the matrix-matched standard solution that achieved a signal-to-noise ratio (S/N) of ≥10. The methodological LOQ was calculated using the following Equation (1):

(1) $$\mathrm{LOQ}\;\left(\mathrm{mg}/\mathrm{kg}\right)=\frac{A\times C}{B\times D}\times E$$

where

*A* = Instrument quantitation limit (ng),
*B* = Sample injection volume (µL),
*C* = Final sample solution volume (mL),
*D* = Amount of analytical sample (g),
*E* = Dilution factor.

The linearity of the calibration curve was evaluated using a seven-point matrix-matched standard calibration method (linear range: 0.005–0.5 mg/kg). To maintain consistency throughout the analysis, the bracket method was applied, incorporating calibration standard solutions at the beginning and end of each sample batch. A regression model was then applied to the calibration data to generate the calibration curve, with the correlation coefficient (*r^2^*) calculated to assess linearity. To assess the recovery of pencycuron, 10 g portions of pesticide-free samples were spiked with 100 µL of standard solutions at concentrations of 1 mg/L and 10 mg/L, resulting in final sample concentrations of 0.01 mg/kg and 0.1 mg/kg, respectively. The recovery samples were subjected to extraction and matrix-matched using the established sample preparation method. After analysis using LC-MS/MS, recovery was determined by comparing the measured concentrations obtained from the calibration curve with the known spiked concentrations. For the storage stability test, samples were treated with the pesticide at a fortification level of 0.1 mg/kg and stored at –20 °C, along with residue samples. After storage, the samples were handled using the identical protocol employed in the recovery assessment. Accuracy was evaluated by analyzing the samples and calculating the recovery rates. The matrix effect was assessed by comparing the slopes of the calibration curves obtained from matrix-matched standard solutions and those from solvent-only standard solutions. Both sets of calibration curves were prepared using the same concentrations. The matrix effect value (ME, %) was calculated using the following Equation (2):

(2) $$\mathrm{ME}\;\left(\%\right)=\left(\frac{\left(Slope\;of\;matrix\;matched\;standard\;calibration\;curve\right)}{\left(Slope\;of\;solvent\;standard\;calibration\;curve\right)}-1\right)\times 100\;\;$$

### 2.7. Estimation of Residue Half-Life

Using the results from residue analysis conducted in the field trials, the residue half-life of pencycuron was calculated, and the PHRL was established. The average residue levels of pencycuron and sampling date were fitted using the regression equation *y* = *ae*^−*bt*^, corresponding to the half-life Equation (3):

(3) $$C_{t}=C_{0}e^{-kt}\;$$

where

*t* is time (d),
*C_t_* is the residue concentration at time (mg/kg),
*C*_0_ is the initial concentration,
*k* is the degradation rate constant.

The degradation rate constant *k* was used to calculate the residue half-life (*t*_1/2_) using Equation (4):

(4) $$t_{1/2}=\frac{ln2}{k}\;$$

### 2.8. Statistical Analysis and Determination of PHRL in Eggplants

To verify the significance of the regression curve (triplicate in each DAT point) at the 95% confidence level, an F-test was conducted using critical values from Snedecor’s F-distribution. The degrees of freedom were determined based on the number of independent variables (numerator degrees of freedom) and the residual error (denominator degrees of freedom). A *t*-test was also performed to assess the significance of the regression coefficient *b* (the degradation rate constant) at the 95% confidence level, using the degrees of freedom associated with the residual error. The 95% confidence interval of the *b* value was calculated using its standard error and the critical value from the *t*-distribution. This confidence interval was used to determine the lower limit of the degradation rate constant (*k*_min_). Using the MRL, the *k*_min_ values for each field and their average, along with the days before harvest (DBH), the PHRL values for each elapsed day were calculated using the following Equation (5):

(5) $$PHRL=MRL\times e^{k_{min}\times DBH}\;\;$$

All statistical computations were performed using Microsoft Excel (Microsoft Corporation, Redmond, WA, USA), version 2410 (build 18129.20116), under a licensed agreement provided by our institution.

## 3. Results and Discussion

### 3.1. Establishment of MRM Conditions for Pencycuron Detection

**Figure 2:**
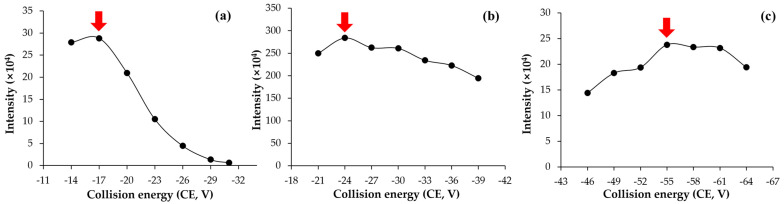
Effect of collision energy (CE) on intensity for (**a**) multiple reaction monitoring (MRM) transition 329.3 to 218.2, (**b**) transition 329.3 to 125.1, and (**c**) transition 329.3 to 89.0. The red arrows in each graph indicate the optimal CE where the highest intensity is observed for each transition.

**Figure 3:**
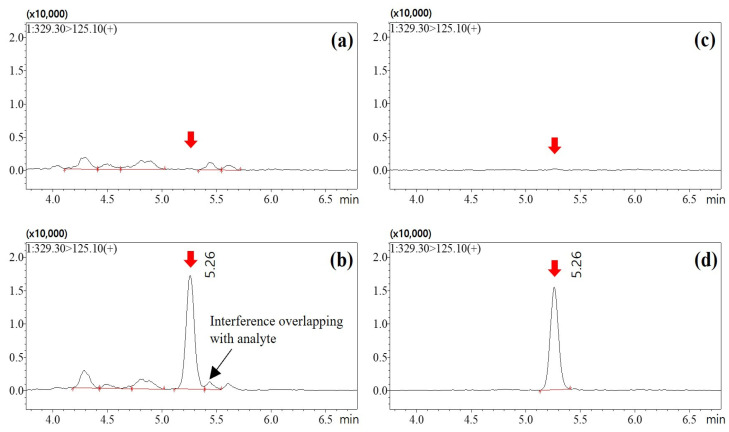
Chromatograms of pencycuron with different dSPE sorbent treatments: (**a**) blank sample using PSA sorbent, (**b**) matrix-matched standard at 0.005 mg/kg using PSA sorbent, (**c**) blank sample using PSA and GCB sorbents, (**d**) matrix-matched standard at 0.005 mg/kg using PSA and GCB sorbents. The red arrows indicate the retention time for pencycuron at 5.26 min.

The multiple reaction monitoring (MRM) conditions for pencycuron were established using LC-MS/MS. Pencycuron was detected in positive ion mode, forming the protonated molecule [M + H]^+^, indicating a proton (H⁺) as an adduct. Through product ion scanning, the major fragment ions were identified as *m*/*z* 125.1, 281.2, and 89.1. The intensities were evaluated under various CE conditions (Figure 2). When the precursor ion fragmented to *m*/*z* 125.1, the highest intensity was observed at CE –24, and when it fragmented to *m*/*z* 218.2, the second highest intensity was observed at CE –17. These ions were selected as the quantifier and qualifier ions, respectively (Table 1). This selection is consistent with the primary MRM transitions observed in the EURL-DataPool data [21]. The transition parameters, particularly the use of *m*/*z* 125 and 218 as the quantifier and qualifier ions, align with established protocols in similar LC-MS/MS analyses of pencycuron [22,23]. The pencycuron reference standard was analyzed under these established MRM conditions, yielding a chromatogram with pencycuron eluting at a retention time (t_R_) of 5.26 min (Table 1 and Figure 3).

### 3.2. Enhanced Purification of Eggplant Crude Extracts with GCB Sorbent

The purification efficiency of GCB treatment was evaluated. Chromatograms of eggplant sample extracts purified solely with PSA as the dSPE sorbent exhibited interferences, indicating incomplete removal of matrix components (Figure 3a). These interferences caused overlapping with the analyte at trace levels near the LOQ (Figure 3b). In contrast, the addition of GCB sorbent (7.5 mg) during the purification process effectively eliminated these interferences, resulting in no analyte overlapping in the chromatogram (Figure 3c,d). These findings demonstrate that GCB is highly effective in purifying the matrix extracted from eggplant samples.

PSA sorbent is known to aid in the elimination of fatty acids, polar organic acids, and various sugars from sample matrices; however, it alone may not sufficiently eliminate interfering substances in complex fruit and highly pigmented vegetable samples [24,25]. GCB, on the other hand, has been shown to effectively remove compounds such as chlorophyll and steroids from extracts [26]. The efficacy of GCB in purifying samples is partly due to its strong affinity for planar molecules, allowing it to adsorb pigments like chlorophyll, carotenoids, and sterols commonly found in foods [27].

Eggplants are characterized by their violet-colored outer skin, attributed to the presence of anthocyanins, while the coloration of the subepidermal cell layers is also influenced by chlorophyll a and b [28]. Both anthocyanins and chlorophyll are planar molecules and are likely to be adsorbed by GCB during the purification process. In contrast, the target analyte, pencycuron, possesses a three-dimensional molecular structure (Figure 1), making it less susceptible to adsorption by GCB. This selective adsorption of interfering planar molecules while retaining the analyte contributes to the high purification efficiency observed when GCB is used.

### 3.3. LC-MS/MS Method Validation for Pencycuron Residues in Eggplant

Method validation subsequently focused on determining the LOQ, linearity, recovery, storage stability, and matrix effect for pencycuron in eggplant using LC-MS/MS (Table 2). The LOQ was defined as the lowest concentration of analyte that could be quantitatively measured with an S/N of 10 or higher [29]. This value was determined to be 0.005 mg per kg sample, calculated using Equation (1).

(1) $$\mathrm{LOQ}\;\left(\mathrm{mg}/\mathrm{kg}\right)=\frac{0.005\;\mathrm{ng}\times 10\;\mathrm{mL}}{2\;\mu L\times 10\;g}\times 2=0.005\;mg/kg$$

According to Codex guidelines—which recommend an LOQ of 0.01 mg/kg or lower for pesticide residues—our method demonstrates enhanced sensitivity and compliance with these standards [30]. Considering that the EU has established the MRL of 0.02 mg/kg for pencycuron in eggplant, the analytical sensitivity of our method is appropriate for regulatory compliance [19]. Earlier HPLC-based methods reported LOQs of 0.02 mg/kg for pencycuron in agricultural products [31]. More recent tandem MS methods have achieved lower LOQs, such as 0.01 mg/kg in agricultural products (GC-MS/MS) [32] and 0.007–0.01 mg/kg when analyzing multiresidues in leafy vegetables (LC-MS/MS) [33,34]. Our method demonstrates enhanced sensitivity, achieving an LOQ lower than those reported in prior studies.

The linearity of the method was evaluated over a concentration range of 0.005 to 0.5 mg/kg for pencycuron in eggplant. The *r^2^* of the calibration curve was found to be 0.999, indicating an excellent linear relationship between the concentration of pencycuron and the detector response within the tested range (Table 2). This high *r^2^* value demonstrates that the method enables precise and dependable quantification of pencycuron throughout the entire calibration range, ensuring its suitability for monitoring trace to moderate levels of the analyte in eggplant samples.

Recovery of the analyte was assessed at fortification levels of 0.01 and 0.1 mg/kg. The recovery rates ranged from 102.6% to 106.1%, with relative standard deviation (RSD) between 2.3% and 6.4% (Table 2). According to the SANTE/11312/2021 guidelines, acceptable recovery ranges from 70% to 120% with an RSD of ≤20% [35], indicating that the obtained results are well within the permissible limits and demonstrate the method’s reliability for accurate quantification of pencycuron in eggplant samples. The excellent recovery can be attributed to pencycuron’s elevated octanol–water partition ratio (K_ow_, log *P* = 4.7), which signifies that pencycuron is more soluble in organic solvents than in water [1,36]. As a result, during the sample preparation, pencycuron preferentially partitions into the acetonitrile layer, the upper organic layer, leading to efficient extraction and higher recovery rates.

To ensure the storage stability of pesticide prior to sample preparation, the accuracy of samples stored for 110 days was evaluated at the 0.1 mg/kg level. The accuracy was determined to be 90.4% with an RSD of 1.0%, confirming that pencycuron remains stable during the storage period. This stability ensures the reliability of the analytical results, even after extended storage times, thereby supporting the method’s robustness of pencycuron in eggplant.

To evaluate the ruggedness of the analytical method, the matrix effect (%ME) of pencycuron was assessed. It was achieved by comparing the slope of the calibration curve in solvent to that in the matrix [37]. A positive percentage (%ME > 0%) indicates signal enhancement, while a negative percentage (%ME < 0%) signifies signal suppression. The %ME was determined to be +8.1%, which falls within the acceptable range of –20% to +20%, indicating a negligible matrix effect [25,38] (Table 2). Compared to studies on leafy vegetables where pencycuron exhibited a matrix effect of –14.5% [33], our method demonstrates a similar level of minimal matrix interference. According to Kmellar et al. (2008), a matrix effect within this range minimizes the risk of false positive or false negative results [38]. This negligible matrix effect ensures accurate quantification of pencycuron, as matrix-induced ionization interferences are minimal. The soft matrix effect observed in our method underscores its reliability and suitability for routine monitoring of pesticide using LC-MS/MS.

### 3.4. Degradation Pattern of Pencycuron Residues in Eggplants

The degradation pattern of pencycuron residues in eggplants was studied over a period of 14 days following the final application (Table 3). The residue concentrations in eggplants showed a decreasing trend over time, starting from an initial residue amount of 0.045 mg/kg immediately after application (0 DAT) and decreasing to 0.006 mg/kg at 14 DAT. Notably, the initial residue amount exceeded the MRL of 0.02 mg/kg [19]. By 3 DAT, the residue level had decreased to 0.020 mg/kg, meeting the MRL. At 5 DAT, the residue level further decreased to 0.016 mg/kg, falling below the MRL, and continued to decrease, reaching near the LOQ (0.005 mg/kg) level at 14 DAT.

**Figure 4:**
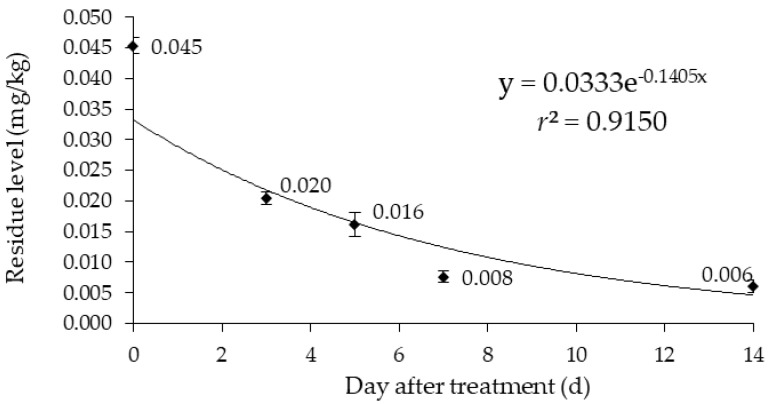
Degradation curve of pencycuron residue in eggplants over time following treatment (triplicate in each DAT point).

The residue levels decreased over time and were best described by a first-order kinetic model (Figure 4). This model is practical for monitoring pesticide residues in crops at harvest according to the pesticide application period and can also be used to screen consumer exposure, making it valuable for risk assessment [13,39]. The fit of the model was evaluated using the coefficient of determination (*r*^2^). The *r*^2^ value obtained was 0.9150 (Figure 4), which is significantly higher than the acceptable threshold of 0.5 [39]. This indicates that this kinetic model adequately explains the decrease in pencycuron residue concentrations over time in eggplants. By finding the degradation rate constant from the regression equation as 0.1405, the half-life (*t*_1/2_) of pencycuron in eggplants was subsequently calculated as 4.9 days (Table 4).

This half-life is longer than the reported half-life of pencycuron in rice paddies, which ranges from 1.6 to 2.8 days [4]. The longer half-life in eggplants may be attributed to the cultivation environment. Greenhouse cultivation in this study has fewer factors contributing to pesticide loss compared to open-field cultivation like rice paddies. Factors such as sunlight exposure and rainfall are limited in greenhouse settings, leading to slower degradation rates. In comparison, the half-lives of other fungicides in eggplants have been reported as 3.3–4.1 d for bifenthrin and 3.5–3.9 d for chlorfenapyr [40]. The differences in half-life among pesticides, even within the same crop, can be attributed to various factors such as formulation types, chemical characteristics of active ingredient, dilution factors of spray solution, and environmental conditions [41].

### 3.5. Proposal of PHRL for Pencycuron in Eggplants

The establishment of the PHRL for pencycuron in eggplants is key to upholding food safety and adhering to regulatory policies [10]. The PHRL was proposed based on the degradation constant (*k*) determined from actual field trials. To ensure the reliability of the proposed PHRL, statistical significance tests were conducted on the regression model and the degradation constant [42]. To evaluate the significance of the first-order kinetic regression model describing the degradation of pencycuron residues, an F-test was performed (Table 4). The calculated F-value was 16.159. This value was evaluated against the critical F-value of 10.128, obtained from Snedecor’s F-distribution with 1 and 5 degrees of freedom at a 95% confidence level. Since the calculated F-value exceeds the critical value (16.159 > 10.128), the regression model established in this study is considered statistically significant at the 95% confidence level. It indicates that the independent variable, time (*t*), has a statistically significant effect on the dependent variable, the residue concentration (*C_t_*).

A *t*-test was performed to evaluate the significance of the degradation rate constant (*k*) (Table 4). The *t*-value for the degradation constant was calculated as 4.020. Since the calculated *t*-value exceeds the critical value (4.020 > 3.182), the degradation constant is statistically significant at the 95% confidence level. This confirms that the degradation constant is a reliable parameter for modeling the residue decline and for establishing the PHRL. The statistical lower limit of the degradation constant, *k*_min_, was calculated by utilizing the lowest estimate from the regression coefficient’s 95% confidence interval. This approach accounts for the slowest possible degradation scenario, assuming the residues decrease at the minimum rate, thereby providing the most conservative estimate for the PHRL [42]. The 95% confidence interval for *k* was calculated as *k* = 0.1405 ± 0.1112. Thus, the lower limit *k*_min_ was 0.0293 (Table 4). Using the MRL of 0.02 mg/kg at 0 DBH [19] and Equation (5), the PHRL values for 10 different days before harvest (DBH) were calculated with both *k* and *k*_min_ (Table 5), resulting in PHRL estimates of 0.03 and 0.08 mg/kg at 10 DBH, respectively. This demonstrates that the choice of degradation constant affects the PHRL estimation, with *k*_min_ yielding a more conservative value. As a result, a more reliable PHRL is established, ensuring that food safety is enhanced by accounting for the slowest possible degradation scenario.

## 4. Conclusions

This study successfully evaluated the residue dynamics of the fungicide pencycuron in eggplants using LC-MS/MS, establishing a reliable method for detection and quantification. The optimized QuEChERS method, combined with d-SPE containing the GCB sorbent, demonstrated high sensitivity and accuracy, with an LOQ of 0.005 mg/kg. Field trials showed that pencycuron residue follows a first-order kinetic degradation model, with a half-life of 4.9 days under greenhouse conditions. Based on these findings, PHRLs were proposed to ensure food safety, accounting for the slowest possible degradation scenario. Overall, the research provides a valuable framework for assessing pesticide residue levels in agricultural produce, supporting regulatory compliance and consumer health protection.

## Figures and Tables

**Table 1:** Multiple reaction monitoring (MRM) conditions and retention time (t_R_) for pencycuron detection in LC-MS/MS.

t_R_ (min)	Monoisotopic Mass	Ionization Type	Precursor Ion to Product Ion (Collision Energy, V)	
			Quantifier Ion	Qualifier Ion
5.26	328.1	[M + H]^+^	329.3 to 125.1 (–24)	329.3 to 218.2 (–17)

**Table 2:** Validation results of the established method for pencycuron analysis in eggplant.

LOQ (mg/kg)	Linearity (*r*^2^)	Recovery, % (RSD ^1^, %)		Storage Stability Accuracy, (RSD, %) at 0.1 mg/kg	Matrix Effect (%)
		0.01 mg/kg	0.1 mg/kg		
0.005	0.999	106.1 (2.3)	102.6 (6.4)	90.4 (1.0)	+8.1

^1^ Relative standard deviation.

**Table 3:** Residue levels of pencycuron in eggplants at various days after treatment (DAT).

DAT (d)	Residue (mg/kg)			
	Trial 1	Trial 2	Trial 3	Mean ± SD
0	0.036	0.040	0.060	0.045 ± 0.013
3	0.022	0.020	0.020	0.020 ± 0.001
5	0.014	0.018	0.016	0.016 ± 0.002
7	0.007	0.007	0.009	0.008 ± 0.001
14	0.006	0.006	0.005	0.006 ± 0.001

**Table 4:** Regression parameters, statistical tests, and confidence intervals for pencycuron residue study.

Regression Equation	Rate Constant (*k*)	Half-Life (*t*_1/2_)	F-Value	F_(1,5;95%)_	*t*-Value	*t*_(5, 0.025)_	Confidence Interval for *k*	*k*_min_
*C_t_* = 0.0333e^−0.1405*t*^	0.1405	4.9	16.159	10.128	4.020	3.182	0.1405 ± 0.1112	0.0293

**Table 5:** Pre-harvest residue limit (PHRL) estimates for pencycuron in eggplants at different days before harvest (DBH) using *k* and *k*_min_.

Rate Constant	Value	PHRL (mg/kg)										
		10 (DBH)	9	8	7	6	5	4	3	2	1	0
*k*	0.1405	0.08	0.07	0.06	0.05	0.05	0.04	0.04	0.03	0.03	0.02	0.02
*k*_min_	0.0293	0.03	0.03	0.03	0.02	0.02	0.02	0.02	0.02	0.02	0.02	0.02

## Data Availability

The original contributions presented in the study are included in the article, further inquiries can be directed to the corresponding author.
